# A DFT Study on Fe^I^/Fe^II^/Fe^III^ Mechanism of the Cross-Coupling between Haloalkane and Aryl Grignard Reagent Catalyzed by Iron-SciOPP Complexes

**DOI:** 10.3390/molecules25163612

**Published:** 2020-08-08

**Authors:** Akhilesh K. Sharma, Masaharu Nakamura

**Affiliations:** 1 International Research Center for Elements Science (IRCELS), Institute for Chemical Research, Kyoto University, Uji, Kyoto 611-0011, Japan; sharma.akhileshkumar.2x@kyoto-u.ac.jp; 2 Department of Energy and Hydrocarbon Chemistry, Graduate School of Engineering, Kyoto University, Kyoto 615-8510, Japan

**Keywords:** iron catalysis, haloalkane coupling, Grignard reagent, Fe^I^/Fe^II^/Fe^III^ mechanism, density functional theory

## Abstract

To explore plausible reaction pathways of the cross-coupling reaction between a haloalkane and an aryl metal reagent catalyzed by an iron–phosphine complex, we examine the reaction of FeBrPh(SciOPP) **1** and bromocycloheptane employing density functional theory (DFT) calculations. Besides the cross-coupling, we also examined the competitive pathways of β-hydrogen elimination to give the corresponding alkene byproduct. The DFT study on the reaction pathways explains the cross-coupling selectivity over the elimination in terms of Fe^I^/Fe^II^/Fe^III^ mechanism which involves the generation of alkyl radical intermediates and their propagation in a chain reaction manner. The present study gives insight into the detailed molecular mechanic of the cross-coupling reaction and revises the Fe^II^/Fe^II^ mechanisms previously proposed by us and others.

## 1. Introduction

Transition-metal-catalyzed cross-coupling reaction is one of the most versatile synthetic tools for constructing carbon frameworks of various functional molecules, e.g., pharmaceuticals, agrochemicals, and organic electronic materials. Iron catalysts have emerged as a sustainable alternative for conventional palladium and nickel catalysts and have attracted significant attention due to their practical merits of cost-effectiveness, low toxicity, and the abundance on the earth [1,2,3,4]. Despite the remarkable progress of iron-catalyzed cross-coupling reactions in organic synthesis [5,6,7,8,9,10,11,12,13,14], their reaction mechanisms remain elusive and attract considerable interests to their underlying molecular mechanisms [15,16,17,18,19].

More specifically, iron catalysts have proven useful for cross-coupling reactions of non-activated haloalkanes, a synthetically valuable yet challenging substrate, which is prone to undergo β-hydrogen elimination in the conventional palladium-catalyzed cross-couplings to form undesired alkene byproducts [20,21,22,23]. Numerous synthetic and mechanistic studies have thus appeared on this class of iron-catalyzed cross-coupling. The use of the controlled-addition technique of Grignard reagent in Kumada–Tamao-Corriu (KTC) coupling or using less reactive boron reagents in Suzuki–Miyaura (SM) coupling have proven, in combination with the use of bulky bisphosphine ligands, to be the successful strategy for the iron-catalyzed haloalkane coupling reactions [24,25,26,27].

**Figure 1:**
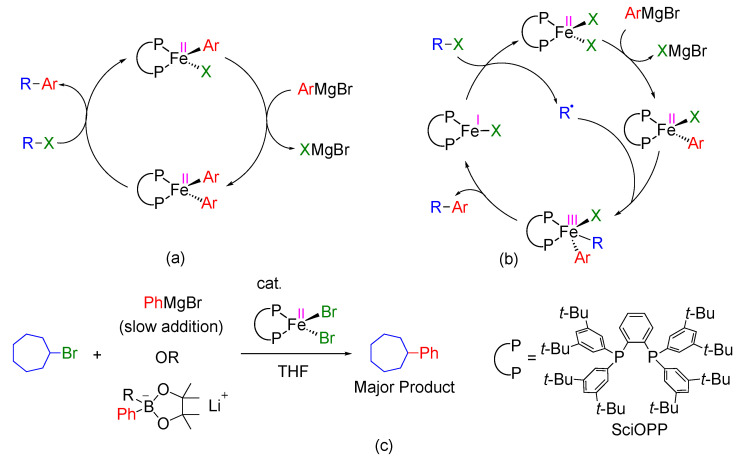
(**a**) Proposed catalytic cycle for cross-coupling between an alkyl halide and Grignard reagent catalyzed by iron-SciOPP complexes. (**b**) Proposed catalytic cycle based on a recent study involving iron-BenzP* catalyzed cross-coupling between α-chloroesters and Grignard reagent. (**c**) The iron-SciOPP-catalyzed cross-coupling reaction chosen for the current density functional theory (DFT) mechanistic study [26,27].

There is a consensus that the haloalkane substrates undergo halogen abstraction by an (organo)iron species to generate the corresponding alkyl radical intermediates. A variety of radical mechanisms have been discussed thus far by Hu [28], Norrby [29], Bedford [30], Tonzetich [31], Fürstner [22], Neidig [32,33], and us [19,34]. Our group and the Neidig group have independently crystalized iron-bisphosphine complexes, such as Fe^II^X_2_(SciOPP), Fe^II^XAr(SciOPP), Fe^II^Ar_2_(SciOPP), where X = halide and Ar = Ph or Mes [32,33,34]. Furthermore, we identified the presence of Fe^II^BrMes(SciOPP), Fe^II^Mes_2_(SciOPP) in the reaction mixture of the cross-coupling reaction by using solution-phase synchrotron X-ray absorption spectroscopic (XAS) studies [34]. According to this study, we proposed an Fe^II^/Fe^II^ mechanism based on identification of Fe^II^Ar_2_(SciOPP) (Figure 1a, Ar = Mes and X = Br). The Neidig group also identified the presence of iron(II) complexes in the solution phase through intensive mechanistic studies, consisting of Mössbauer and magnetic circular dichroism (MCD) spectroscopy, and synthetic approaches [32,33]. They identified that Fe^II^XAr(SciOPP) is the active iron species of the cross-coupling reaction suppressing undesired β-hydrogen elimination reactions (Figure 1a, Ar = Ph). On the other hand, the Bedford group crystalized iron(I) species, [Fe^I^Br(dpbz)_2_] which is formed from iron(II)-precatalyst and they proposed a low-coordinate iron(I)-phosphine species is involved in the cross-coupling reactions [35]. Identification of such low-coordinate iron(I)-complexes in the reaction mixture was not fully successful, and their involvement in the reaction mechanism remains elusive. The active iron-species that generate alkyl radical intermediates and the iron-species that react with the radical intermediate to effect the carbon–carbon bond formation have also remained unknown.

Recently, our group developed enantioselective cross-coupling reactions using chiral bisphosphine ligands and proposed that the radical species may not react with the iron(III)-species which takes part in the generation of the radical (in-cage mechanism) [36,37]. We further performed the detailed DFT analysis of the reaction of α-chloroesters with the Grignard reagent in presence of chiral bisphosphine ligand and found that Fe^I^/Fe^II^/Fe^III^ mechanism operates in the reaction (Figure 1b) [38]. The iron(I) species activates the carbon–halogen bond of halo-ester substrates leading to the generation of α-ester-radical. In the next step, another molecule of Fe^II^XAr (active species) traps the generated radical (out-of-cage or chain mechanism) and lead to product formation through iron(III) species. Parallelly, the Gutierrez group reported DFT mechanistic study of the same reaction leading to the virtually same conclusion [39]. Despite these recent mechanistic insights, the use of electronically and sterically different iron-bisphosphine complexes, different substrates, and reagents in cross-coupling reactions make it difficult to generalize the mechanism understanding.

The iron-SciOPP catalysts have been known as a superior catalyst for the cross-coupling of alkyl halides and Grignard reagent [26]. Due to bulky nature and strong chelation ability of the ligand, it avoids the formation of multinuclear or multiligand iron–phosphine complexes and also suppresses the formation of undesired ate complexes, which presumably responsible for increased reactivity and selectivity by minimizing side reactions. The mechanistic studies by Neidig showed that Fe^II^BrPh(SciOPP) complex reacts with a haloalkane to give the corresponding cross-coupling product selectively, but the detailed mechanism has remained unclear [33]. In our pursuit to identify the iron species involved in the elementary steps of the iron-catalyzed haloalkane coupling, DFT is used to study the reaction between Fe^II^BrPh(SciOPP) and bromocycloheptane (Figure 1c), which has been believed as the key for the selective cross-coupling of a haloalkane with an aryl metal reagent in the presence of an iron-bisphosphine catalyst. The plausible reaction pathways, Fe^II^/Fe^II^, and Fe^I^/Fe^II^/Fe^III^ are examined and compared in this study.

## 2. Results and Discussion

### 2.1. Reaction of Fe^II^BrPh(SciOPP) with Bromocycloheptane

#### 2.1.1. Cycloheptyl Radical Generation via C–Br Activation by Iron(II)-Species

**Figure 2:**
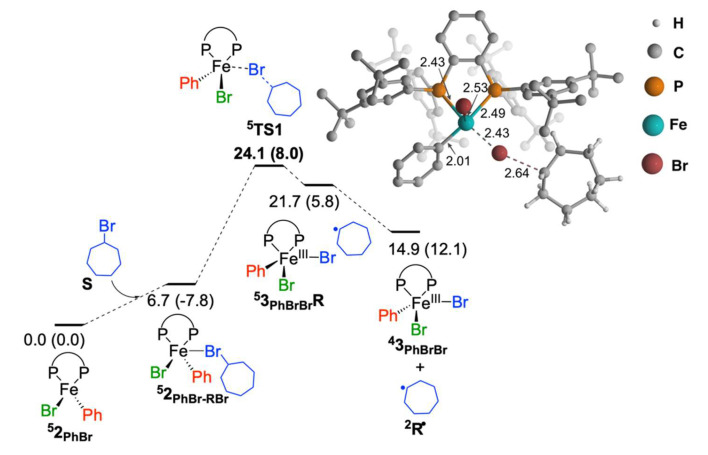
Free energy profile for the reaction of iron(II) species with bromocycloheptane. The relative free energies with respect to **^5^2_PhBr_** and bromocycloheptane are given in kcal/mol with total electronic energies including ZPE (zero-point energy) correction (ΔE_ZPE_) are given in parenthesis.

The reaction mechanism from Fe^II^BrPh(SciOPP) is studied as it is predicted to be the starting species in cross-coupling reactions. The TSs with lowest energy spin states as found in our previous study were only considered, due to large system size [38]. The first step is the C–Br activation of bromocycloheptane by quintet Fe^II^BrPh(SciOPP) (**^5^2_PhBr_**), through an atom transfer mechanism (Figure 2). To a tetrahedral geometry of Fe^II^BrPh(SciOPP), **^5^2_PhBr_**, bromine-atom of substrate (**S**) approaches in the plane the same as P–Fe–P to form pre-reaction complex **^5^2_PhBr-RBr_**, which is 6.7 kcal/mol higher in energy than the separated molecules. The **^5^TS1** has a distorted square-pyramidal geometry, with the incoming bromine-atom positions in the square plane and the other bromine in the pyramidal position. The activation barrier of this process is 24.1 kcal/mol. The Mulliken spin densities on the iron center of the catalyst, and C_1_ and bromine of substrate are ρ(Fe) = 3.316, ρ (Br) = −0.055, and ρ(C_1_) = 0.672, respectively (Figure S1), indicating that during this step, quintet iron(II) is converted to quartet-iron(III) species with the generation of doublet cycloheptyl radical, **^2^R·**. The formed **^5^3_PhBrBr_R** and **^4^3_PhBrBr_** species are respectively 21.7 and 14.9 kcal/mol higher in energy than **^5^2_PhBr_**, suggesting the reversible nature of the C–Br activation and will lead to a low concentration of radical **^2^R·**.

#### 2.1.2. Reaction of Cycloheptyl Radical with Iron(II)-Species: C–C Bond Formation and Generation of Iron(I) Species

**Figure 3:**
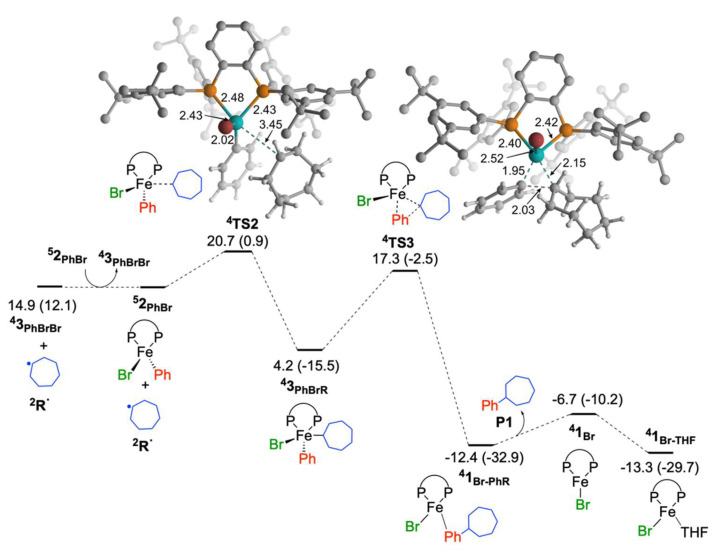
Free energy profile for cross-coupling by the reaction of iron(II) species with cycloheptyl radical, where radical is generated by the reaction of iron(II) species and bromocycloheptane. Free energies with respect to **^5^2_PhBr_** and bromocycloheptane are given in kcal/mol (ΔE_ZPE_ are given in parenthesis).

The generated radical intermediate **R·** can react with either **^4^3_PhBrBr_** (iron(III)-species, in-cage mechanism) or **^5^2_PhBr_** (iron(II)-species, out-of-cage mechanism) present in the reaction mixture [36,38]. For the reaction of cycloheptyl radical (**R·**) with iron(II) species, the first step involves the out-of-cage movement of **^2^R·** from the intermediate **^4^3_PhBrBr_R**. Then the **^2^R·** coordinates to the iron center through **^4^TS2** with a lower barrier than **^5^TS1** (20.7 kcal/mol from starting **^5^2_PhBr_** and 5.8 kcal/mol from the second **^5^2_PhBr_**, Figure 3). It leads to the formation of lower energy iron(III) species **^4^3_PhBrR_**, which is 11.7 kcal/mol lower in energy. The consequent reductive elimination forms C–C coupling product **P1**, which proceeds with the activation barrier of 13.1 kcal/mol (**^4^TS3**). The reductive elimination process is highly exergonic and the corresponding iron(I)-product complex (**^4^1_Br-PhR_**) is 16.6 kcal/mol lower in energy than **^4^3_PhBrR_**. Afterwards, the product will decoordinate from **^4^1_Br-PhR_** leading to the formation of 5.7 kcal/mol higher energy species **^4^1_Br_**, probably through a barrierless process. The transient species **^4^1_Br_** readily reacts with a THF molecule (solvent) leading to the formation of lower energy species **^4^1_Br-THF_** (0.9 kcal/mol lower energy than **^4^1_Br-PhR_**). The whole process for the reaction of Fe^II^BrPh(SciOPP) with bromocycloheptane leading to the formation of product and **^4^1_Br-THF_** is exergonic by 13.3 kcal/mol.

#### 2.1.3. Reaction of Cycloheptyl Radical with Fe^III^Br_2_Ph(SciOPP) (In-Cage Mechanism)

The reaction of cycloheptyl radical with **^4^3_PhBrBr_** leading to a cross-coupling product turns out to have a higher free energy barrier of 26.9 kcal/mol (Figure S2). This process occurs through an outer-sphere fashion (**^5^TS4**), where **R·** attacks directly the *ipso*-carbon of the phenyl group and does not coordinate with the iron center. The reaction leading to byproduct alkene formation occurs by the simultaneous transfer of bromine and hydrogen from bromocycloheptane to iron and the *ipso*-carbon of the phenyl group of **^5^2_PhBr_**, respectively, via **^5^TS5** with a 25.2 kcal/mol energy barrier. Both processes for the formation of the cross-coupling and the alkene products are highly exothermic (ca. 35–45 kcal/mol). The formation of alkene is favorable kinetically over the cross-coupling. However, it does not agree with experimental observation.

Although the reaction of cycloheptyl radical with Fe^III^Br_2_Ph(SciOPP), **^4^3_PhBrBr_** leading to cross-coupling product and alkene formation is highly exothermic, it is unlikely as **^5^TS4** and **^5^TS5** are 6.2 and 4.5 kcal/mol higher in energy than **^4^TS2**. Hence, the cross-coupling reaction proceeds by the reaction of alkyl radical with iron(II)-species. Similar to our previous study, iron-SciOPP complexes also do not favor Fe^II^/Fe^III^ mechanism.

#### 2.1.4. Reaction of Cycloheptyl Radical with Iron(II)-Species: Alkene Byproduct Formation

**Figure 4:**
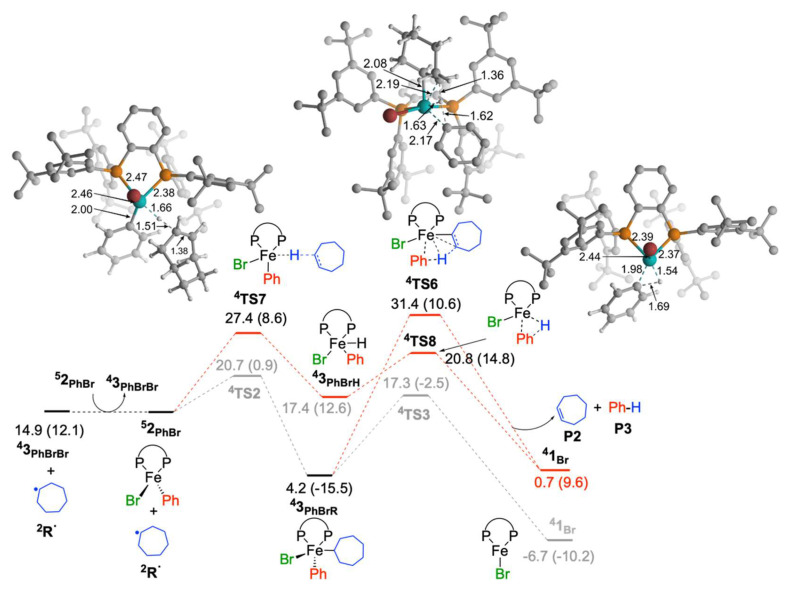
Free energy profile for olefin formation by reaction of Fe^II^ species with cycloheptyl radical, where radical is generated by the reaction of iron(II) species and bromocycloheptane. Free energies with respect to **^5^2_PhBr_** and bromocycloheptane are given in kcal/mol (ΔE_ZPE_ are given in parenthesis).

From complex **^4^3_PhBrR_** the possibility of formation of alkene is also considered (Figure 4). It involves the transfer of β-hydrogen from a coordinated cycloheptyl group to phenyl ligand (**^4^TS6**). The activation barrier for this process is apparently high and **^4^TS6** is 14.1 kcal/mol higher in energy than **^4^TS3** and the formation of alkene is unlikely from **^4^3_PhBrR_**. The TSs for coupling and alkene formation by the outer-sphere reaction of radical with phenyl of iron(II) species, **^5^2_PhBr_** could not be located.

Further, we checked the possibility of alkene formation by H-atom transfer (**^4^TS7**) from cycloheptyl radical to iron-atom of the iron(II) complex, **^5^2_PhBr_**. The activation barrier for this process is 12.9 kcal/mol. It leads to the formation of the iron(III)-hydride complex, **^4^3_PhBrH_**. The following reductive elimination between the hydride and phenyl groups through **^4^TS8** lead to the formation of benzene. This pathway is unfavorable as **^4^TS7** is 7.7 kcal/mol higher in energy than **^4^TS2**. Hence, the reaction of the alkyl radical with Fe^II^BrPh(SciOPP) does not give the alkene byproduct, which is in concert with the experimental observations.

#### 2.1.5. Reaction of Iron(I) Species with Bromocycloheptane: Regeneration of Fe^II^Br_2_(SciOPP) and Cycloheptyl Radical

**Figure 5:**
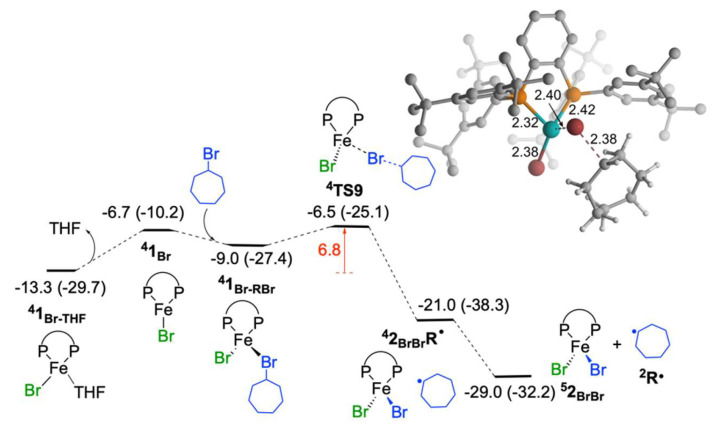
Free energy profile for probable reaction path of generated iron(I) species. Free energies with respect to **^5^2_PhBr_** and bromocycloheptane are given in kcal/mol (ΔE_ZPE_ are given in parenthesis).

The iron(I) species formed during the reaction can react either with another iron(III) species to undergo a comproportionation to generate two iron(II) species, or with haloalkane substrate (of which concentration is larger than iron(III) species) to generate one iron(II) species and an alkyl radical intermediate. For the reaction to proceed the first step is the removal of THF from **^4^1_Br-THF_** to form **^4^1_Br_** and this process is endergonic by 6.6 kcal/mol (Figure 5). This step is required for both comproportionation and reaction with the haloalkane substrate. The coordination of the substrate through bromide to **^4^1_Br_**, leading to the formation of **^4^1_Br-BrR_** is exergonic by 2.3 kcal/mol. The activation barrier for the Br-atom transfer (**^4^TS9**) from **^4^1_Br-BrR_** species is 6.8 kcal/mol (barrier from **^4^1_Br-THF_**). The process is highly exergonic and leads to formation of cycloheptyl radical and iron(II) species **^5^2_BrBr_**. The generated **^5^2_BrBr_** species will undergo transmetalation and the radical species will react with the available **^5^2_PhBr_** in the reaction mixture, propagating the radical chain reaction [40]. Hence, the reaction will proceed through the *out-of-cage* mechanism, and similar to our previous study, the current reaction also follows the Fe^I^/Fe^II^/Fe^III^ pathway [38].

The comproportionation between **^4^1_Br_** and **^4^3_PhBrBr_** (generated by substrate reaction with iron(II) species **^5^2_PhBr_**) may be a barrierless process and is a highly exergonic process (37.2 kcal/mol). Since the concentration of the haloalkane substrate is high enough under the catalytic coupling condition, the reaction between the iron(I) species and haloalkane is more likely. Hence, iron(I) species is the active species after its generation during the reaction, which reduces the activation barrier for C–Cl activation from 24.1 to 6.8 kcal/mol.

### 2.2. Proposed Catalytic Cycle Based on Fe^I^/Fe^II^/Fe^III^ Mechanism

**Figure 6:**
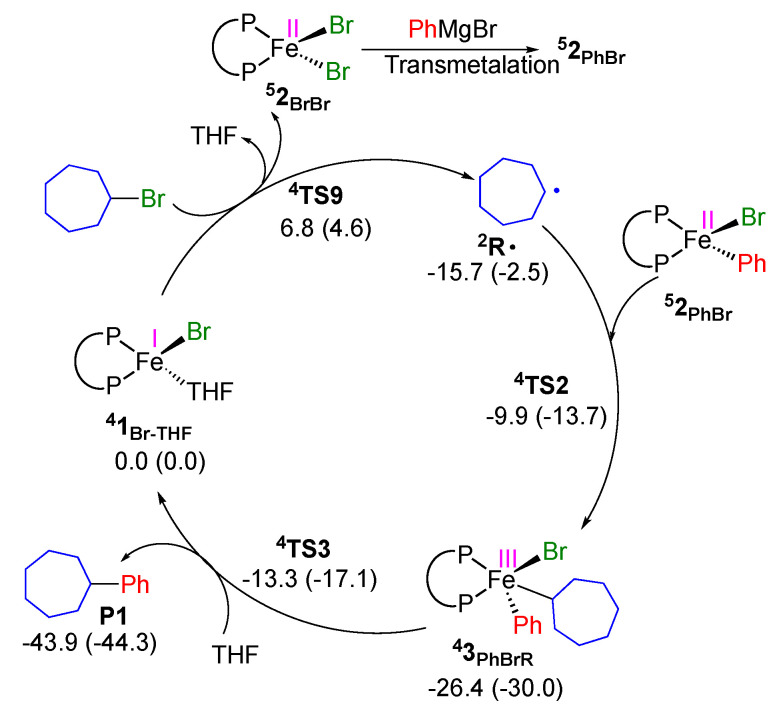
Revised catalytic cycle of iron-SciOPP catalyzed-KTC coupling of a haloalkane based on the current DFT study. Free energies with respect to **^4^1_Br-THF_** and bromocycloheptane are given in kcal/mol (ΔE_ZPE_ are given in parenthesis).

Based on the above theoretical studies, we propose the reaction mechanism depicted in Figure 6. The Fe^II^XAr(SciOPP) (**^5^2_PhBr_**) is first converted to Fe^I^XTHF(SciOPP) (**^4^1_Br-THF_**) with the formation of the cross-coupling product. Afterwards, iron(I) species (**^4^1_Br-THF_**) mainly activate the alky halide. Transmetalation of **^4^1_Br-THF_** is less likely due to the low concentration of Grignard reagent, which excludes the possibility of involvement of Fe^I^ArTHF(SciOPP) species. Hence, the catalytic cycle starts with the halogen atom abstraction from the alkyl halide (Alkyl-X) by iron(I) species (**^4^1_Br_**) affording a dihaloiron species Fe^II^X_2_(SciOPP) (**^5^2_BrBr_**) along with an alkyl radical intermediate (**^2^R·**) (Figure 6 and Figure S3). The resultant alkyl radical **^2^R·** escapes from the solvent cage and is further trapped by iron(II) species, Fe^II^XAr(SciOPP) (**^5^2_PhBr_**) (which have a higher concentration) via the out-of-cage pathway. It led to the formation of iron(III) species Fe^III^XArR(SciOPP) (**^4^3_PhBrR_**). The reductive elimination from **^4^3_PhBrR_** led to the formation of a cross-coupling product and regenerating iron(I) species (**^4^1_Br-THF_**). Based on this mechanism, the detection of both iron(II) and iron(I) species, and their roles in the cross-coupling reaction can be reasonably explained.

## 3. Computational Methods

The Gaussian09 program was used for quantum mechanical calculations reported in the study [41]. The DFT method B3LYP [42,43] was used for geometry optimization and energy calculations with the inclusion of solvent effects by the PCM method [44]. Grimme’s D3 dispersion correction method was used to include dispersion correction [45]. For geometry optimization, the 6-31G* basis set [46] was used for all atoms as it gave good agreement with the crystal structure of *Fe^II^BrPh(SciOPP)* and *Fe ^II^Br_2_(SciOPP)* (Figure S4). The geometry optimization with SDD [47] basis set for Fe, Br and 6-31G* for other atoms lead to relatively longer metal ligand distances. Hence, the 6-31G* basis set was used. The hessian calculation was performed to confirm minima (no imaginary frequency) and TS (one imaginary frequency). All TS were confirmed by visual inspection of imaginary frequency. The connectivity between TSs and minima were confirmed by IRC [48] calculations for 20 steps and subsequent optimization of the geometry at PCM/B3LYP/6-31G* level of theory.

The energies were further computed using the 6-311G** basis set [49] for all atoms and with Douglas–Kroll–Hess second-order scalar relativistic effects. The E_ZPE_ reported in the paper is calculated by adding zero-point-energy obtained at the 6-31G* basis set to electronic energy of higher basis set (E_ZPE_ = E_elec(6-311G**)_ + ZPE_(6-31G*)_). The free energies were calculated by adding thermal free energy correction obtained at 6-31G* to electronic energy of the 6-311G** basis set (G = E_elec(6-311G**)_ + G_Corr(6-31G*)_). The free energies were calculated at 298.15 K temperature and 1 atm pressure. Concisely, the energies reported in the paper were calculated at PCM_THF_/B3LYP-D3/6-311G**//PCM_THF_/B3LYP-D3/6-31G* level of theory.

The spin-state energies of iron complexes computed by a DFT method depend on the choice of the exchange-correlation functional [50,51]. Various functionals were recommended in the literature, while no consensus has been made for the optimal choice of the DFT method. Hence, care should be taken in choosing functional, especially for iron complexes with the small spin-state energy gaps. The DFT method (B3LYP-D3 Functional) used in this work predicted quintet as the ground state for iron(II) complexes, Fe^II^BrPh(SciOPP) and Fe^II^Br_2_(SciOPP), which is in agreement with the experimentally known spin state [33,34]. Additionally, the spin state for related iron(I) complex (Fe(η^2^-[TIPS-CC-H])Br(SciOPP)) was also predicted correctly [52]. Further, the OBPE-D3 functional (which is known to provide reliable spin state gap) [53] gave consistent results with B3LYP-D3 for the ground spin state (quartet) of iron(I) and iron(III) complexes (Table S1). The OPBE-D3 functional failed to give reliable results for the spin state of the Fe^II^BrPh(SciOPP) complex. Hence, the B3LYP-method reliably predicts the spin state of the current system and is used in this study. Further, we have also checked the effect of long-range-corrected (LC) exchange functional on barrier height [54] using CAM-B3LYP-D3 functional. The CAM-B3LYP-D3 functional led to a similar conclusion as of B3LYP-D3, and some TSs showed a higher energy barrier (details are given in Table S2).

## 4. Conclusions

We have studied the mechanism of the reaction between Fe^II^BrPh(SciOPP) and bromocycloheptane by using DFT methods and identified iron species of different oxidation states involved in a catalytic cycle of the iron-bisphosphine-catalyzed haloalkane couplings. The reaction mechanism follows the Fe^I^/Fe^II^/Fe^III^ pathway, where iron(I) activates the C–Br bond of the haloalkane substrate, another molecule of Fe^II^BrPh(SciOPP) traps the generated alkyl radical species to form iron(III) species leading to the formation of the cross-coupling product and iron(I) species. The iron(I) species then again react with haloalkane generating alkyl radical and the reaction propagates through a radical chain mechanism. The current mechanism mirrors our previous mechanistic study on a chiral bisphosphine-catalyzed enantioselective cross-coupling despite the change of bisphosphine ligand from BenzP* to SciOPP.

The reaction of haloalkane with iron(II) species and the reaction of the alkyl radical with iron(III) species have a higher energy barrier than those of Iron(I) species and iron(II) species, respectively. The obtained energetics exclude the possibility of Fe^II^/Fe^II^ and Fe^II^/Fe^III^ pathways proposed previously. Furthermore, we have found that an alkyl radical prefers to coordinate to the Fe^II^BrPh(SciOPP) species, instead of the β-hydrogen-atom abstraction ending with the alkene byproduct. The generated Fe^III^BrPh(alkyl)(SciOPP) species favors reductive elimination to give the corresponding coupling product over the alkene byproduct. These results deepen our understanding of the iron-SciOPP catalysis and provide insights into the probable root for the side product formations. Further understanding of the mechanism of alkene formation with Fe^II^Ph_2_(SciOPP) catalyst will help design better catalysts and synthetic methodology. The theoretical and experimental research in this line is now actively pursued in our laboratory and will be reported in due course.

## Supplementary Materials

The following are available online. Cartesian coordinates of optimized geometries and their energies, Figure S1: Mulliken spin densities of the stationary points, Figure S2: Free energy profile for the reaction of Fe^II/^Fe^III^ mechanism, Figure S3: Energy profile for Fe^I^/Fe^II^/Fe^III^ pathway starting the reaction from iron(I) species, Figure S4: Comparison of geometrical parameters of X-ray crystal structure of **2_PhBr_** and **2_BrBr_** with the optimized geometry in quintet spin state, Table S1: The comparison of energies for the iron(I), iron(II) and iron(III) complexes in different spin states at B3LYP-D3 and OPBE-D3 functionals. Table S2: Comparison of free energy barriers of catalytic cycle at B3LYP-D3 and CAM-B3LYP-D3 functionals.

## References

[1] Guðmundsson A., Bäckvall J.-E. (2020). On the use of iron in organic chemistry. Molecules.

[2] Fürstner A. (2016). Iron catalysis in organic synthesis: A critical assessment of what it takes to make this base metal a multitasking champion. ACS Cent. Sci..

[3] Bauer I., Knölker H.-J. (2015). Iron catalysis in organic synthesis. Chem. Rev..

[4] Bolm C., Legros J., Le Paih J., Zani L. (2004). Iron-catalyzed reactions in organic synthesis. Chem. Rev..

[5] Piontek A., Bisz E., Szostak M. (2018). Iron-catalyzed cross-couplings in the synthesis of pharmaceuticals: In pursuit of sustainability. Angew. Chem. Int. Ed..

[6] Legros J., Figadere B. (2015). Iron-promoted C–C bond formation in the total synthesis of natural products and drugs. Nat. Prod. Rep..

[7] Cahiez G., Knochel P., Kuzmina O., Steib A., Moyeux A. (2015). Recent advances in iron-catalyzed Csp2–Csp2 cross-couplings. Synthesis.

[8] Ilies L., Nakamura E. (2012). Iron-catalyzed cross-coupling reactions. PATAI’S Chem. Funct. Groups.

[9] Guérinot A., Cossy J. (2016). Iron-catalyzed C–C cross-couplings using organometallics. Top. Curr. Chem..

[10] Tamura M., Kochi J.K. (1971). Vinylation of grignard reagents. Catalysis by iron. J. Am. Chem. Soc..

[11] Reddy C.K., Knochel P. (1996). New cobalt and iron-catalyzed reactions of organozinc compounds. Angew. Chem. Int. Ed..

[12] Cahiez G., Avedissian H. (1998). Highly stereo and chemoselective iron-catalyzed alkenylation of organomagnesium compounds. Synthesis.

[13] Fürstner A., Leitner A., Méndez M., Krause H. (2002). Iron-catalyzed cross-coupling reactions. J. Am. Chem. Soc..

[14] Hatakeyama T., Nakamura M. (2007). Iron-catalyzed selective biaryl coupling: Remarkable suppression of homocoupling by the fluoride anion. J. Am. Chem. Soc..

[15] Bedford R.B. (2015). How low does iron go? Chasing the active species in Fe-catalyzed cross-coupling reactions. Acc. Chem. Res..

[16] Neidig M.L., Carpenter S.H., Curran D.J., DeMuth J.C., Fleischauer V.E., Iannuzzi T.E., Neate P.G.N., Sears J.D., Wolford N.J. (2018). Development and evolution of mechanistic understanding in iron-catalyzed cross-coupling. Acc. Chem. Res..

[17] Parchomyk T., Koszinowski K. (2017). Iron-catalyzed cross-coupling: Mechanistic insight for rational applications in synthesis. Synthesis.

[18] Mako T.L., Byers J.A. (2016). Recent advances in iron-catalysed cross coupling reactions and their mechanistic underpinning. Inorg. Chem. Front..

[19] Noda D., Sunada Y., Hatakeyama T., Nakamura M., Nagashima H. (2009). Effect of TMEDA on iron-catalyzed coupling reactions of ArMgX with Alkyl halides. J. Am. Chem. Soc..

[20] Nakamura M., Matsuo K., Ito S., Nakamura E. (2004). Iron-catalyzed cross-coupling of primary and secondary Alkyl halides with Aryl grignard reagents. J. Am. Chem. Soc..

[21] Nagano T., Hayashi T. (2004). Iron-catalyzed grignard cross-coupling with Alkyl halides possessing β-hydrogens. Org. Lett..

[22] Martin R., Fürstner A. (2004). Cross-coupling of Alkyl halides with Aryl grignard reagents catalyzed by a low-valent iron complex. Angew. Chem. Int. Ed..

[23] Bedford R.B., Bruce D.W., Frost R.M., Goodby J.W., Hird M. (2004). Iron(III) salen-type catalysts for the cross-coupling of aryl grignards with alkyl halides bearing β-hydrogens. Chem. Commun..

[24] Bedford R.B., Huwe M., Wilkinson M.C. (2009). Iron-catalysed Negishi coupling of benzylhalides and phosphates. Chem. Commun..

[25] Hatakeyama T., Kondo Y., Fujiwara Y.-I., Takaya H., Ito S., Nakamura E., Nakamura M. (2009). Iron-catalysed fluoroaromatic coupling reactions under catalytic modulation with 1,2-bis (diphenylphosphino) benzene. Chem. Commun..

[26] Hatakeyama T., Hashimoto T., Kondo Y., Fujiwara Y., Seike H., Takaya H., Tamada Y., Ono T., Nakamura M. (2010). Iron-catalyzed Suzuki−Miyaura coupling of Alkyl halides. J. Am. Chem. Soc..

[27] Hatakeyama T., Fujiwara Y.-I., Okada Y., Itoh T., Hashimoto T., Kawamura S., Ogata K., Takaya H., Nakamura M. (2011). Kumada–Tamao–Corriu coupling of Alkyl halides catalyzed by an iron–bisphosphine complex. Chem. Lett..

[28] Bauer G., Wodrich M.D., Scopelliti R., Hu X. (2014). Iron pincer complexes as catalysts and intermediates in Alkyl–Aryl Kumada coupling reactions. Organometallics.

[29] Hedström A., Izakian Z., Vreto I., Wallentin C.-J., Norrby P.-O. (2015). On the radical nature of iron-catalyzed cross-coupling reactions. Chem. A Eur. J..

[30] Bedford R.B., Brenner P.B., Richards E., Carvell T.W., Cogswell P.M., Gallagher T., Harvey J., Murphy D., Neeve E.C., Nunn J. (2014). Expedient iron-catalyzed coupling of Alkyl, Benzyl and Allyl halides with Arylboronic esters. Chem. A Eur. J..

[31] Przyojski J.A., Veggeberg K.P., Arman H.D., Tonzetich Z.J. (2015). Mechanistic studies of catalytic carbon–carbon cross-coupling by well-defined iron NHC complexes. ACS Catal..

[32] Daifuku S.L., Al-Afyouni M.H., Snyder B.E.R., Kneebone J.L., Neidig M.L. (2014). A combined Moössbauer, magnetic circular dichroism, and density functional theory approach for iron cross-coupling catalysis: Electronic structure, In Situ Formation, and reactivity of iron-Mesityl-Bisphosphines. J. Am. Chem. Soc..

[33] Daifuku S.L., Kneebone J.L., Snyder B.E.R., Neidig M.L. (2015). Iron (II) active species in iron–bisphosphine catalyzed Kumada and Suzuki–Miyaura cross-couplings of Phenyl Nucleophiles and secondary Alkyl halides. J. Am. Chem. Soc..

[34] Takaya H., Nakajima S., Nakagawa N., Isozaki K., Iwamoto T., Imayoshi R., Gower N.J., Adak L., Hatakeyama T., Honma T. (2015). Investigation of organoiron catalysis in Kumada–Tamao–Corriu-type cross-coupling reaction assisted by solution-phase X-ray absorption spectroscopy. Bull. Chem. Soc. Jpn..

[35] Adams C.J., Bedford R.B., Carter E., Gower N.J., Haddow M., Harvey J., Huwe M., Cartes M.Á., Mansell S.M., Mendoza C. (2012). Iron(I) in Negishi cross-coupling reactions. J. Am. Chem. Soc..

[36] Jin M., Adak L., Nakamura M. (2015). Iron-catalyzed enantioselective cross-coupling reactions of α-chloroesters with Aryl grignard reagents. J. Am. Chem. Soc..

[37] Iwamoto T., Okuzono C., Adak L., Jin M., Nakamura M. (2019). Iron-catalysed enantioselective Suzuki–Miyaura coupling of racemic alkyl bromides. Chem. Commun..

[38] Sharma A.K., Sameera W.M.C., Jin M., Adak L., Okuzono C., Iwamoto T., Kato M., Nakamura M., Morokuma K. (2017). DFT and AFIR study on the mechanism and the origin of enantioselectivity in iron-catalyzed cross-coupling reactions. J. Am. Chem. Soc..

[39] Lee W., Zhou J., Gutierrez O. (2017). Mechanism of Nakamura’s Bisphosphine-iron-catalyzed asymmetric C(sp2)–C(sp3) cross-coupling reaction: The role of spin in controlling Arylation pathways. J. Am. Chem. Soc..

[40] Schley N.D., Fu G.C. (2014). Nickel-catalyzed Negishi Arylations of Propargylic bromides: A mechanistic investigation. J. Am. Chem. Soc..

[41] Frisch M.J., Trucks G.W., Schlegel H.B., Scuseria G.E., Robb M.A., Cheeseman J.R., Scalmani G., Barone V., Mennucci B., Petersson G.A. (2009). Gaussian 09, Revision D.01/E.01.

[42] Becke A.D. (1988). Density-functional exchange-energy approximation with correct asymptotic behavior. Phys. Rev. A.

[43] Lee C., Yang W., Parr R.G. (1988). Development of the Colle-Salvetti correlation-energy formula into a functional of the electron density. Phys. Rev. B.

[44] Tomasi J., Mennucci B., Cammi R. (2005). Quantum mechanical continuum solvation models. Chem. Rev..

[45] Grimme S., Antony J., Ehrlich S., Krieg H. (2010). A consistent and accurate ab initio parametrization of density functional dispersion correction (DFT-D) for the 94 elements H-Pu. J. Chem. Phys..

[46] Ditchfield R. (1971). Self-consistent molecular-orbital methods. IX. An extended Gaussian-type basis for molecular-orbital studies of organic molecules. J. Chem. Phys..

[47] Dolg M., Wedig U., Stoll H., Preuss H. (1987). Energy-adjusted ab initio pseudopotentials for the first row transition elements. J. Chem. Phys..

[48] Fukui K. (1981). The path of chemical reactions—The IRC approach. Acc. Chem. Res..

[49] Krishnan R., Binkley J.S., Seeger R., Pople J.A. (1980). Self-consistent molecular orbital methods. XX. A basis set for correlated wave functions. J. Chem. Phys..

[50] Radoń M. (2019). Benchmarking quantum chemistry methods for spin-state energetics of iron complexes against quantitative experimental data. Phys. Chem. Chem. Phys..

[51] Siig O.S., Kepp K.P. (2018). Iron(II) and Iron(III) spin crossover: Toward an optimal density functional. J. Phys. Chem. A.

[52] Kneebone J.L., Brennessel W.W., Neidig M.L. (2017). Intermediates and reactivity in iron-catalyzed cross-couplings of Alkynyl grignards with Alkyl Halides. J. Am. Chem. Soc..

[53] Swart M. (2008). Accurate spin-state energies for iron complexes. J. Chem. Theory Comput..

[54] Song J.-W., Hirosawa T., Tsuneda T., Hirao K. (2007). Long-range corrected density functional calculations of chemical reactions: Redetermination of parameter. J. Chem. Phys..

