# Supplementary material for: A DFT Study on FeI/FeII/FeIII Mechanism of the Cross-Coupling between Haloalkane and Aryl Grignard Reagent Catalyzed by Iron-SciOPP Complexes
Source: Molecules. 2020 Aug 8;25(16):3612. doi: 10.3390/molecules25163612 (PMC7465158; doi:10.3390/molecules25163612)
Supplement: Supplementary file 1 [file molecules-25-03612-s001.zip › molecules-869756supp/molecules-869756supp.pdf]

Supplementary Information

A DFT Study on $Fe^{I}/Fe^{II}/Fe^{III}$ Mechanism of the Cross Coupling between Haloalkane and Aryl Grignard Reagent Catalyzed by Iron-SciOPP Complexes

Akhilesh K. Sharma[1] and Masaharu Nakamura[1,2,*]

[1] International Research Center for Elements Science (IRCELS), Institute for Chemical Research, Kyoto University, Uji, Kyoto 611-0011, Japan
[2] Department of Energy and Hydrocarbon Chemistry, Graduate School of Engineering, Kyoto University, Kyoto 615-8510, Japan
* Correspondence: masaharu@scl.kyoto-u.ac.jp

Content

Figure S1. Mulliken spin densities of the stationary points.

Figure S2. Free energy profile for the reaction of Fe^{II}/Fe^{III} mechanism.

Figure S3. Energy profile for $Fe^{I}/Fe^{II}/Fe^{III}$ pathway starting the reaction from iron(I) species.

Figure S4. Comparison of geometrical parameters of X-ray crystal structure of $\mathbf{2}_{PhBr}$ and $\mathbf{2}_{BrBr}$ with the optimized geometry in quintet spin state.

Table S1. The comparison of energies for the iron(I), iron(II) and iron(III) complexes in different spin states at B3LYP-D3 and OPBE-D3 functionals.

Table S2. The comparison of free energies for the stationary points at B3LYP-D3 and CAM-B3LYP-D3 functionals.

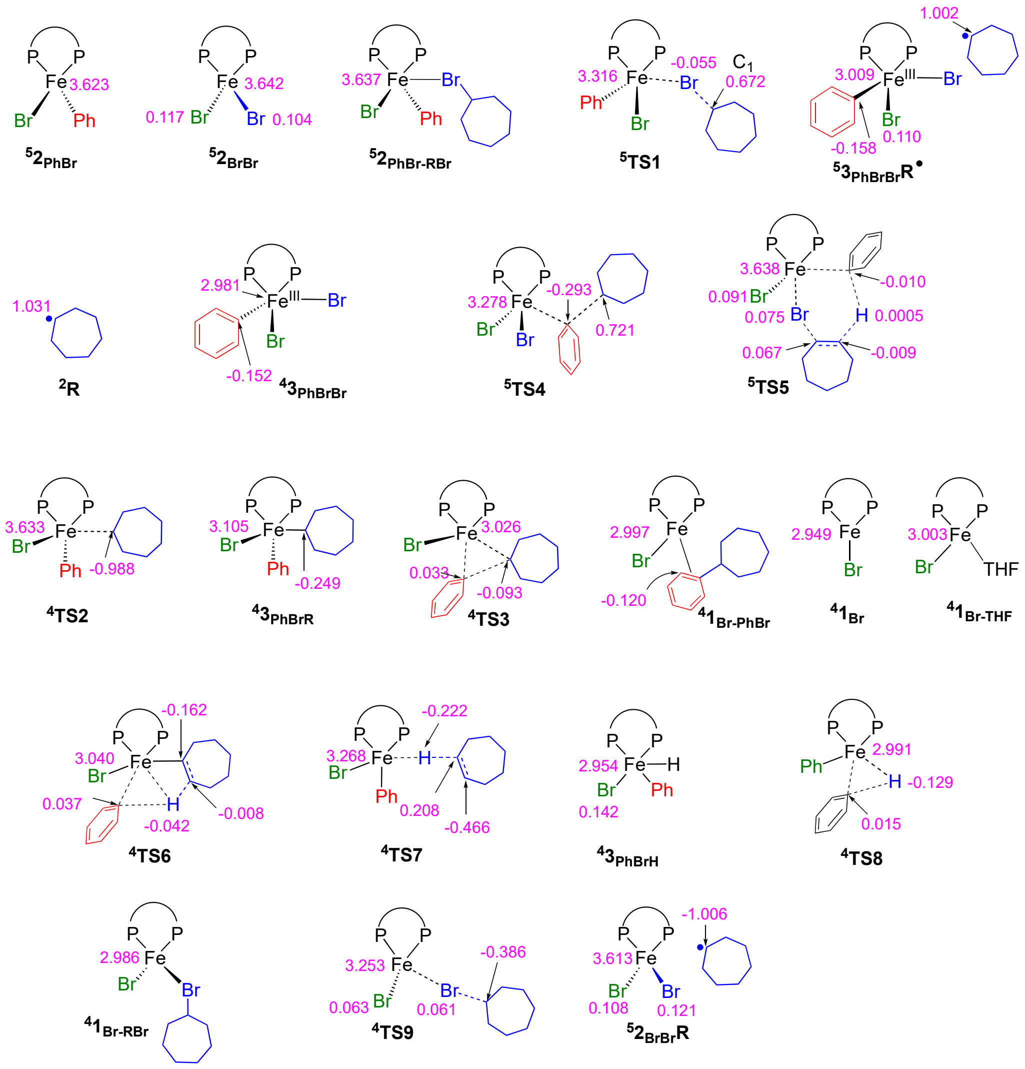

Figure S1. Mulliken spin densities of the stationary points. Only spin densities >0.1 are given (except for the atoms involved in reaction coordinate). (PCMTHF/B3LYP-D3/6-311G**//PCMTHF/B3LYP-D3/6-31G*).

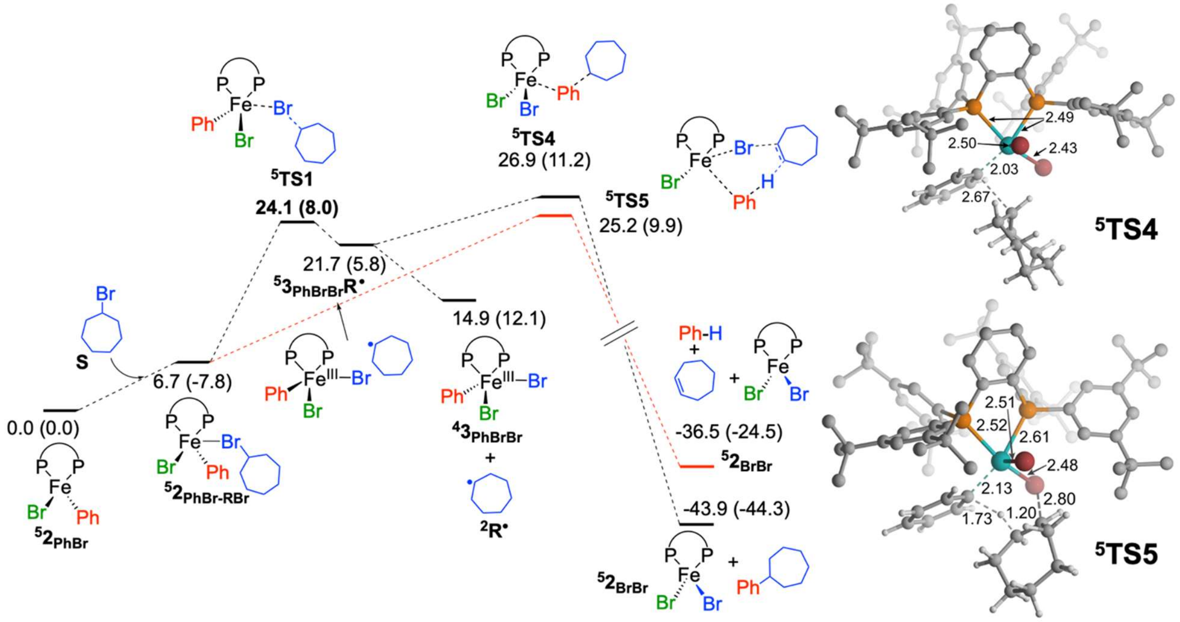

Figure S2. Free energy profile for the reaction of Fe^{II}/Fe^{III} mechanism for cross-coupling reaction. The pathway for the alkene formation is also shown. Free energies are given in kcal/mol with total electronic energy including ZPE-correction is given in parenthesis. (PCM_{THF}/B3LYP-D3/6-311G**//PCM_{THF}/B3LYP-D3/6-31G*).

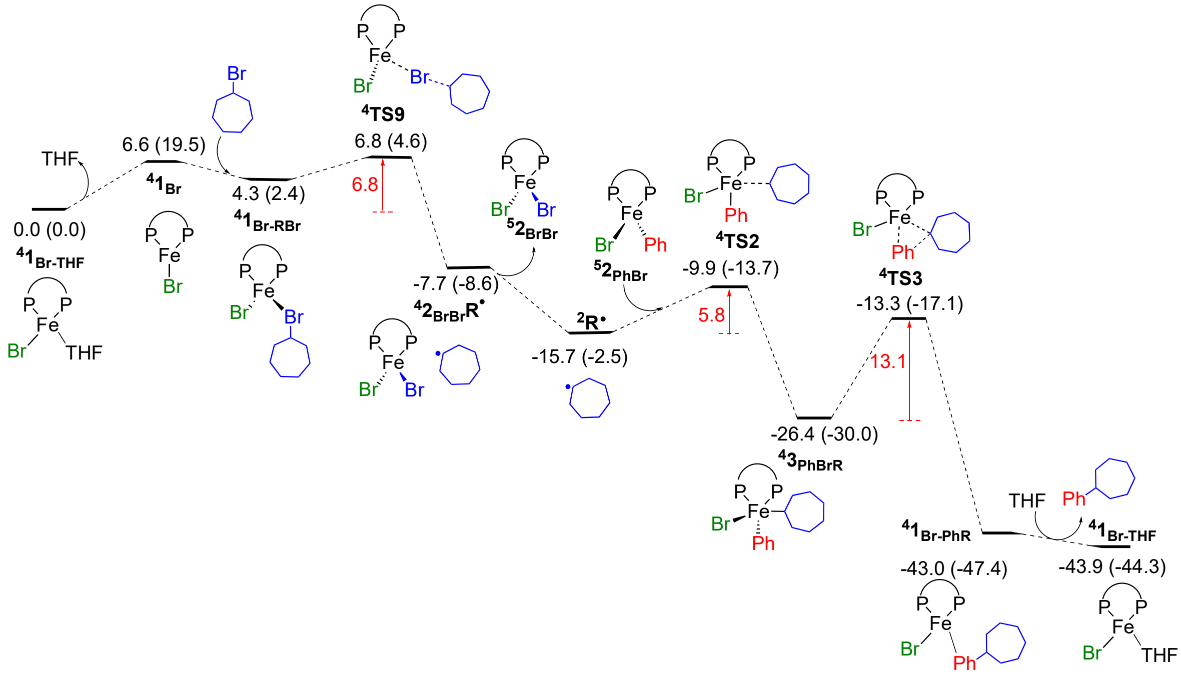

Figure S3. Energy profile for $Fe^{I}/Fe^{II}/Fe^{III}$ pathway starting the reaction from iron(I) species. Free energies are given in kcal/mol with total electronic energy including ZPE-correction is given in parenthesis. (PCM_{THF}/B3LYP-D3/6-311G**//PCM_{THF}/B3LYP-D3/6-31G*).

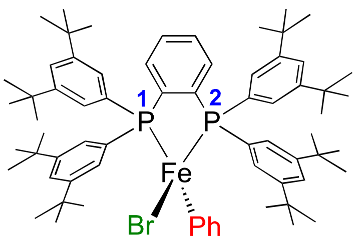

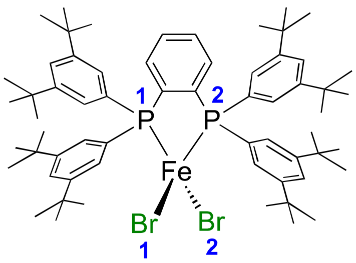

	Crystal	L1	L2	L3	L4
d(Fe-P$_1$)	2.42	2.42	2.47	2.46	2.46
d(Fe-P$_2$)	2.44	2.47	2.50	2.50	2.50
d(Fe-C$_{Ph}$)	2.07	2.03	2.04	2.04	2.04
d(Fe-Br)	2.38	2.40	2.44	2.46	2.49
∠(P$_1$-Fe-P$_2$)	80.5	79.5	79.5	79.6	79.8
∠(Br-Fe-C$_{Ph}$)	114.1	125.3	127.8	126.3	125.3

(a)

	Crystal	L1
d(Fe-P$_1$)	2.43	2.43
d(Fe-P$_2$)	2.46	2.48
d(Fe-Br$_1$)	2.37	2.39
d(Fe-Br$_2$)	2.35	2.37
∠(P$_1$-Fe-P$_2$)	81.47	80.34
∠(Br$_1$-Fe-Br$_2$)	117.3	127.2

(b)

L1 = PCM$_{THF}$/B3LYP-D3/6-31G*
L2 = PCM$_{THF}$/B3LYP-D3/SDD(Fe),6-31G*(other atoms)
L3 = PCM$_{THF}$/B3LYP-D3/SDD(Fe),6-31+G*(P,Br),6-31G*(C,H)
L4 = PCM$_{THF}$/B3LYP-D3/SDD(Fe,Br),6-31G*(other atoms)

Figure S4. (a) Comparison of selected geometrical parameters of X-ray crystal structure of $\mathbf{2}_{\mathbf{PhBr}}$ and optimized quintet spin state ($^{5}\mathbf{2}_{\mathbf{PhBr}}$) geometry at different level of theories (L1-L4). (b) Comparison of selected geometrical parameters of X-ray crystal structure of $\mathbf{2}_{\mathbf{BrBr}}$ and optimized quintet spin state ($^{5}\mathbf{2}_{\mathbf{BrBr}}$) geometry at L1 level of theory. All bond lengths are given in Å and angles are given in degree.

Table S1. The comparison of energies for the iron(I), iron(II) and irin(III) complexes in different spin states at B3LYP-D3 and OPBE-D3 functionals (L1-L3).

	L1 (B3LYP) [a]		L2 (OPBE) [a]		L3 (OPBE) [a]	
	ΔEzpe	ΔG	ΔEzpe	ΔG	ΔEzpe	ΔG
Fe(II)BrPh(SciOPP) (Td or SqP) [b]						
$^{1}2_{PhBr}$ (Td)	17.9	21.0	-9.2	-6.0	-12.3	-9.6
$^{1}2_{PhBr}$ (SqP)	24.1	25.2	14.7	15.8	13.2	14.4
$^{3}2_{PhBr}$ (Td)	16.3	17.6	2.3	3.6	0.1	1.0
$^{3}2_{PhBr}$ (SqP)	11.6	11.8	-0.9	-0.7	-0.3	0.8
$^{5}2_{PhBr}$ (Td)	0.0	0.0	0.0	0.0	0.0	0.0
$^{5}2_{PhBr}$ (SqP)	-[c]	-[c]	-[c]	-[c]	18.8	17.9
Fe(II)Br2(SciOPP) (Td or SqP) [b]						
$^{1}2_{PhBr}$ (Td)	28.6	30.1	22.9	24.4		
$^{1}2_{PhBr}$ (SqP)	33.0	33.1	25.9	26.0		
$^{3}2_{PhBr}$ (Td)	21.9	23.1	10.6	11.7		
$^{3}2_{PhBr}$ (SqP)	20.4	19.3	10.2	9.1		
$^{5}2_{PhBr}$ (Td)	0.0	0.0	0.0	0.0		
$^{5}2_{PhBr}$ (SqP)	-[c]	-[c]	-[c]	-[c]		
Fe(I)Br(SciOPP)(R-Ph)						
$^{2}1_{Br\text{-}PhR}$	21.6	21.5	15.4	15.3		
$^{4}3_{Br\text{-}PhR}$	0.0	0.0	0.0	0.0		
$^{6}3_{Br\text{-}PhR}$ [e]	33.2	32.4	40.1	39.3		
Fe(I)Br(SciOPP)(THF)						
$^{2}1_{Br\text{-}THF}$	18.3	18.9	9.1	9.7		
$^{4}1_{Br\text{-}THF}$	0.0	0.0	0.0	0.0		
$^{6}1_{Br\text{-}THF}$ [e]	27.5	28.0	31.8	32.3		
Fe(η2-[TIPS-CC-H])Br(SciOPP) [d]						
$^{2}1_{Br\text{-}Alkyne}$	16.6	17.4	9.7	10.6		

$^{4}1_{Br\text{-}Alkyne}$	0.0	0.0	0.0	0.0
Fe(III)BrPh(SciOPP)(R)				
$^{2}3_{PhBrR}$	18.3	20.7	13.4	15.8
$^{4}3_{PhBrR}$	0.0	0.0	0.0	0.0
$^{6}3_{PhBrR}$	18.0	16.6	25.1	23.7
Fe(III)Br2Ph(SciOPP)				
$^{2}3_{PhBrBr}$	15.6	16.6		
$^{4}3_{PhBrBr}$	0.0	0.0		
$^{6}3_{PhBrBr}$	8.8	7.6		
Fe(III)BrHPh(SciOPP)				
$^{2}3_{PhBrH}$	23.5	25.2		
$^{4}3_{PhBrH}$	0.0	0.0		
$^{6}3_{PhBrH}$	18.7	18.5		

[a] L1 = PCM_{THF}/B3LYP-D3/6-311G**//PCM_{THF}/B3LYP-D3/6-31G*); L2 = PCM_{THF}/OPBE-D3/6-311G**//PCM_{THF}/B3LYP-D3/6-31G* and L3 = PCM_{THF}/OPBE-D3/6-311G**//PCM_{THF}/OPBE-D3/6-31G*. [b] Both tetrahedral (Td) and square planar geometries are considered for iron (II) complexes. [c] The guess of square planar geometry converged to tetrahedral geometry. Hence, the current stationary point could not be obtained. Also, it is known to be higher in energy as evident from energy at L3 level of theory for $^{5}2_{PhBr}$ (SqP). [d] As there is no information available in literature about spin state of Fe(I) complex, the related iron (I) complex Fe(η2-[TIPS-CC-H])Br(SciOPP) (**$1_{Br\text{-}Alkyne}$**) whose crystal structure and spin state (S=3/2) was recently reported by Neidig and coworkers was used to identify performance of B3LYP functional. [e] Although the sextet (S=5/2) spin state is not feasible for d^7 iron(I) complex, these are considered as the reductive elimination from sextet iron(III) complex can generate such species (if kinetically feasible).

Table S2. The comparison of free energies [a] for the stationary points at B3LYP and CAM-B3LYP functionals.

	**B3LYP-D3**	**CAM-B3LYP-D3**	**B3LYP-D3**	**CAM-B3LYP-D3**
	Relative Gibbs free energies (ΔG)		*Free energy Barrier*	
**$^{4}1_{Br\text{-}THF}$**	0.0	0.0		
**$^{4}1_{Br}$**	6.6	7.8		
**$^{4}1_{Br\text{-}RBr}$**	4.3	6.4		
**^{4}TS9**	6.8	13.0	6.8	13.0
**$^{4}2_{BrBr}R^{\bullet}$**	-7.7	-8.1		
**$^{2}R^{\bullet}$**	-15.7	-17.7		
**^{4}TS2**	-9.9	-10.2	5.8	7.5
**$^{4}3_{PhBrR}$**	-26.4	-23.9		
**^{4}TS3**	-13.3	-10.9	13.1	13.0
**$^{4}1_{Br\text{-}PhR}$**	-43.0	-43.5		
**$^{4}1_{Br\text{-}THF}$**	-43.9	-46.7		
**^{4}TS6**	0.8	2.8	27.2	26.7
**^{4}TS7**	-3.3	1.5	23.2	25.4
**$^{5}2_{PhBr}$** [b]	0.0	0.0	0.0	0.0
**^{5}TS1** [b]	24.1	33.4	24.1	33.4
**^{5}TS4** [b]	26.9	35.6	26.9	35.6
**^{5}TS5** [b]	25.2	32.7	25.2	32.7

[a] The relative energies are with respect to **$^{4}1_{Br\text{-}THF}$** and bromocycloheptane. The energies are calculated with 6-311G** basis set and PCM implicit solvation method using geometry optimized at PCM_{THF}/B3LYP-D3/6-31G* level of theory. [b] The relative energies are calculated using with respect to **$^{5}2_{PhBr}$** and bromocycloheptane.

Note: The B3LYP functional has been shown to underestimate the reaction barrier heights in a benchmark study (*J. Chem. Phys.* **2007**, *126*, 154105) and long-range-corrected DFT methods were proposed to give better results. Hence, we tested the effect of LC functional, CAM-B3LYP on the

activation barrier. We found a large change in barrier heights for steps involving the iron(I) (**TS9**) and iron(II) (**TS1**, **TS4**, and **TS5**) (see Table S2 above). The **TS1** has a higher barrier (33.4 kcal/mol) at CAM-B3LYP functional. Although the step involving **TS1** is required only during the initiation of the reaction, the higher barrier will slow down reaction considerably and seemingly not matching with experimental results, where Kumada-Tamao-Corriu Coupling reaction proceeds at 25°C and giving product in 20 minutes. Further, CAM-B3LYP results do not change any conclusion of the paper, except for the higher barrier. Hence, despite the limitations of the B3LYP functional used, at current juncture it seems to be a good method, considering more reliable double-hybrid-DFT and *ab-initio* methods are computationally not viable for large system.
